# Pentatricopeptide repeat proteins in crops: Advances in functional mechanisms and breeding applications

**DOI:** 10.1111/jipb.70217

**Published:** 2026-03-19

**Authors:** Mingming Wu, Maohong Cai, Rongrong Zhai, Jing Ye, Guofu Zhu, Faming Yu, Shenghai Ye, Xiaoming Zhang

**Affiliations:** ^1^ Institute of Crop and Nuclear Technology Utilization, Zhejiang Academy of Agricultural Sciences Hangzhou 310021 China; ^2^ College of Life and Environmental Science Hangzhou Normal University Hangzhou 311121 China

**Keywords:** CMS, organelle RNA processing, pentatricopeptide repeat (PPR), photosynthesis, seed development

## Abstract

Pentatricopeptide repeat (PPR) proteins constitute a large superfamily of nuclear‐encoded proteins characterized by tandem helical repeats. They function as critical coordinators of nucleus‐organelle communication by modulating RNA metabolism within chloroplasts and mitochondria. This review summarizes recent advances in understanding the functional mechanisms of PPR proteins in major cereal and oilseed crops, with a focus on their roles in regulating seedling growth, stress responses, seed development, and cytoplasmic male sterility (CMS) restoration. We highlight how chloroplast‐localized PPR proteins mediate RNA metabolism to ensure proper chloroplast biogenesis and seedling photosynthesis, while mitochondrial‐targeted PPR proteins are crucial for RNA processing events that support respiration, embryo and endosperm development, and fertility restoration in CMS systems. We also describe how certain PPR proteins mediate biotic and abiotic stress responses through their functions in cold, drought, salt, and disease resistance, with specific members localized in chloroplasts or mitochondria. Finally, we outline unresolved questions regarding PPR protein complex assembly and environmental modulation, and highlight the emerging potential of engineered designer PPR (dPPR) proteins as programmable tools for precise RNA targeting and manipulation in organelles.

## Graphical abstract

Pentatricopeptide repeat proteins coordinate nucleus‐organelle communication by modulating RNA metabolism within chloroplasts and mitochondria. This review highlights how they control critical processes like photosynthesis, seed development, fertility restoration, and stress survival in crops, and explores their potential as programmable tools for RNA manipulation in organelles.

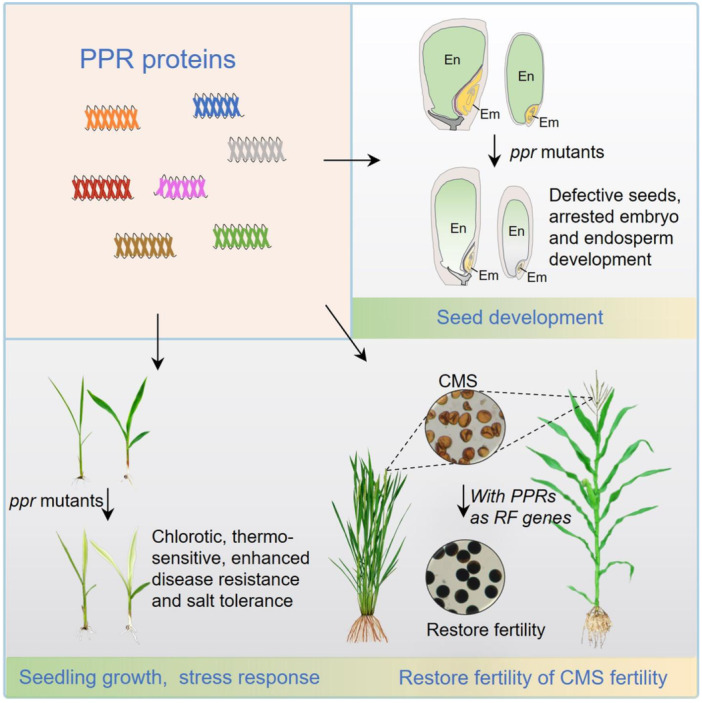

## INTRODUCTION

Human survival is intricately linked to the advancement of crops. Seeds, in particular, serve as vital repositories for the products of photosynthesis occurring in leaves. Cereal crop seeds, including wheat (*Triticum aestivum*), rice (*Oryza sativa*), and maize (*Zea mays*), along with essential oilseed species such as rapeseed (*Brassica napus*), constitute the primary nutritional sources for humans and livestock. The synthesis and storage of carbohydrates within the leaves and seeds, as well as zygote formation, lay the foundation for the survival and successive generation of crops. Mitochondria and chloroplasts are integral to enabling these critical metabolic functions.

Phylogenetic evidence indicates that mitochondria and chloroplasts originated from two endosymbiotic events in primitive eukaryotes, with mitochondria originating from an ancestral α‐proteobacterial symbiont and chloroplasts evolving from endosymbiotic cyanobacteria (Gould et al., 2008; Nunnari and Suomalainen, 2012; Keeling, 2013). Through co‐evolution with eukaryotic hosts, these organelles experienced extensive genome reduction via gene transfer to the nuclear genome. This evolutionary process established a complex regulatory framework in which nuclear‐encoded factors govern organellar functions (Timmis et al., 2004; Bock, 2017; Raval et al., 2023). In angiosperms, the residual organellar genomes exhibit dramatically reduced sizes compared with their nuclear counterparts. Nevertheless, the precise regulation of organellar RNA processing, including stability, editing, splicing, cleavage, and translation, remains critical for maintaining photosynthetic efficiency in chloroplasts and oxidative phosphorylation in mitochondria (Small et al., 2023; Zhang et al., 2023c). These processes are particularly vital during reproductive development and seed formation, where coordinated intercompartmental signaling between nuclear and organellar systems ensures proper biosynthesis of structural components and energy metabolism enzymes. Accumulating studies have highlighted the central regulatory role played by PPR proteins, a large family of nuclear‐encoded proteins, in mediating these intricate processes (Bolter and Soll, 2016; Wiedemann and Pfanner, 2017; Small et al., 2023).

Since being discovered more than two decades ago, the PPR protein family has been systematically characterized via Arabidopsis genome sequencing (Aubourg et al., 2000; Small and Peeters, 2000; Lurin et al., 2004). PPR proteins constitute a large family of highly specific RNA‐binding proteins, widely diversified across eukaryotes (Schmitz‐Linneweber and Small, 2008). While their canonical function is RNA recognition via PPR motifs, functional studies reveal a spectrum of mechanisms, with emerging evidence indicating that some members may perform roles beyond mere binding (Wang et al., 2021a; Jiang et al., 2022; Lin et al., 2024; Song et al., 2024). After completion of genome sequencing for major crops such as rice and maize, the PPR gene family has been identified in crops, and extensive research has been conducted on these PPR genes (O'Toole et al., 2008; Fujii and Small, 2011; Liu et al., 2016a; Chen et al., 2018a; 2018b; Su et al., 2019). Numerous studies have shown that PPR proteins play roles in crop growth and development. Ten years ago, Alice et al. summarized and reviewed the molecular functions of PPRs, mainly in Arabidopsis and maize (Barkan and Small, 2014). In the past decade, considerable progress has been achieved in the identification and functional characterization of PPR proteins. Therefore, summarizing and reviewing the literature on PPR proteins is helpful for in‐depth studies of PPR protein functions in different species. Recently, the cellular functions and action mechanisms of PPR proteins within plants were reviewed (Wang and Tan, 2024). In this review, we summarize the research progress and applications of PPR proteins in crops over the past decade, with a focus on their roles in photosynthesis, seed development, fertility restoration, and stress responses. Furthermore, we highlight the emerging potential of engineered designer PPR proteins as programmable RNA‐targeting tools for precise gene manipulation and breeding applications.

## STRUCTURAL AND FUNCTIONAL DIVERSITY OF PPR PROTEINS

The PPR gene family represents one of the largest nuclear‐encoded gene families in angiosperms, comprising over 450 members in the model plant Arabidopsis, and approximately 400 members in major cereal crops such as rice and maize (Lurin et al., 2004; O'Toole et al., 2008; Chen et al., 2018a; 2018b). Members of the PPR family in cereal crops, like those in Arabidopsis, typically consist of a single coding exon, with most localized in mitochondria and/or chloroplasts, and a smaller number reported to be localized in the nucleus or cytoplasm (Lurin et al., 2004; Barkan and Small, 2014; Hao et al., 2019; Xue et al., 2019; Zheng et al., 2022). Structurally, PPR proteins generally contain N‐terminal subcellular localization signal peptides, followed by 2–30 tandem repeats of the PPR motif, each defined by a canonical 35 amino acids (Schmitz‐Linneweber and Small, 2008; Barkan and Small, 2014). PPR proteins are divided into P‐type and PLS‐type subfamilies. The P‐type subgroup exclusively contains canonical PPR (P) motifs, while PLS‐type members display an ordered arrangement of three distinct motifs (P, L, and S) differing in length (Barkan and Small, 2014). Functional divergence between these subclasses is further reflected in their C‐terminal domain architectures. Some P‐type proteins may possess a small MutS‐related (SMR) domain conferring RNA endonuclease activity, whereas PLS‐type members typically contain conserved E, E + , and DYW domains, and are called PPR‐E, PPR‐E + , and PPR‐DYW proteins (Schmitz‐Linneweber and Small, 2008; Barkan and Small, 2014; Wu et al., 2016b; Zhou et al., 2017). PPR‐E proteins contain only the RNA‐binding PPR motifs and a C‐terminal E domain, functioning primarily as sequence‐specific recognition scaffolds. PPR‐E+ proteins possess an additional E+ domain following the E domain, which enables them to specifically recruit downstream editing enzymes. PPR‐DYW proteins feature the complete set of E, E + , and DYW domains. Notably, the DYW domain of PPR‐DYW proteins possesses intrinsic cytidine deaminase activity capable of directly catalyzing C‐to‐U conversion, as evidenced by crystal structure analysis of Arabidopsis PPR‐DYW protein OTP86, which revealed a structure similar to bacterial deaminase *Ec*CD and confirmed its catalytic function (Takenaka et al., 2021; Wang et al., 2023).

**Figure 1:**
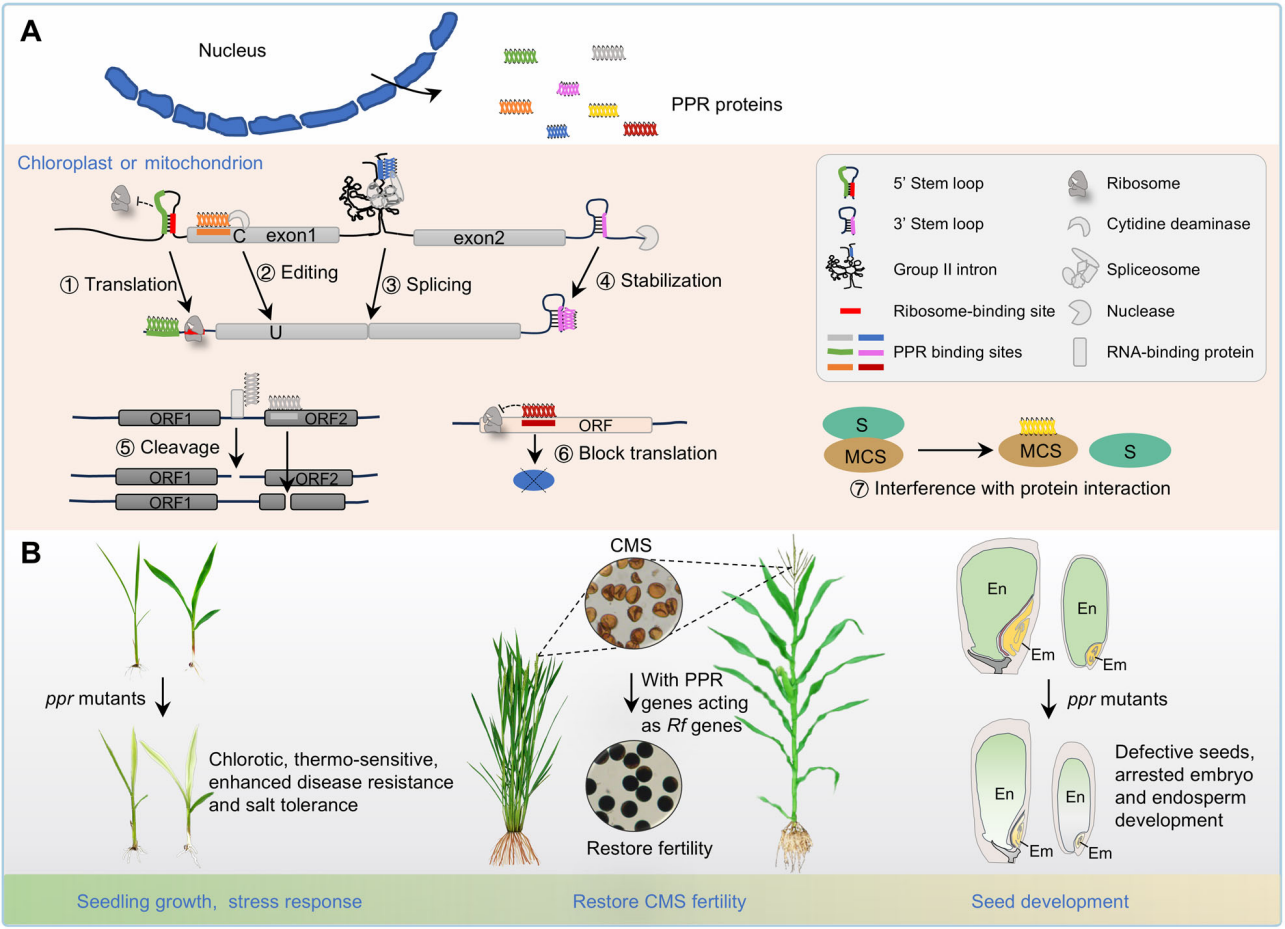
Regulatory roles of PPR proteins in crop development and stress responses **(A)** PPR proteins encoded by nuclear genes are predominantly localized within chloroplasts or mitochondria. Within these organelles, they play a pivotal role in regulating diverse RNA metabolism processes. These functions of PPR proteins encompass the following aspects: ① Translation. PPR proteins bind to RNA to prevent the formation of secondary structures that mask the ribosome‐binding site (RBS), thereby facilitating translation initiation. ② Editing. PPR proteins, either those containing a cytidine deaminase DYW domain or those that form an editosome complex, facilitate site‐specific Cytidine‐to‐Uridine (C‐to‐U) RNA editing. ③ Splicing. PPR proteins bind to specific sequences at introns or exon‐intron junctions to modulate spliceosome assembly, thereby enabling the precise splicing of pre‐mRNAs. ④ Stabilization. PPR proteins bind to specific RNA sequences, protecting transcripts from nuclease degradation and enhancing RNA stability. ⑤ Cleavage. PPR proteins can bind to their targets or form a complex to perform precise cleavage at specific RNA sites. ⑥ Block translation. PPR proteins bind to key regions of mRNAs, creating steric hindrance that directly impedes ribosome binding or progression, thereby inhibiting translation. ⑦ Interference with protein interactions. PPR proteins can interfere with complex‐dependent ROS production by disrupting critical protein‐protein interactions. S represents a sterility‐related protein, and MCS denotes a mitochondrial complex subunit. **(B)** Schematic depiction of PPR protein functions in crops. Left panel, *ppr* mutants exhibit thermo‐sensitive chlorotic phenotypes and altered stress responses (enhanced disease resistance/salt tolerance) during seedling growth. Middle panel, PPR act as Rf genes to restore fertility in cytoplasmic male sterile (CMS). Right panel, *ppr* mutants cause defective seed development, with arrested embryo (Em) and endosperm (En) development. These observations collectively demonstrate the key roles of PPR proteins in seedling growth, stress responses, fertility restoration, and seed development.

Despite the extraordinary size and ubiquitous distribution of PPR proteins in plants, they perform specialized functions (Figure 1A). P‐type PPRs primarily regulate RNA metabolism in organelles through a diverse set of processes: facilitating transcript splicing, mediating RNA stabilization, promoting RNA cleavage, regulating translational control (including both translation initiation and blockage), and interfering with protein interactions (Hu et al., 2012; Barkan and Small, 2014; Melonek et al., 2021; Jiang et al., 2022; Lin et al., 2024; Song et al., 2024). In contrast, PLS‐type members orchestrate RNA editing via editosome complexes with auxiliary factors such as multi‐organelle RNA editing factors, organelle RNA recognition motif‐containing proteins, protoporphyrin IX oxidase 1, and organelle zinc finger proteins (Takenaka et al., 2012; Sun et al., 2013; Zhang et al., 2014). The structural elucidation of PPR proteins has revealed conserved architectural principles, in which each PPR motif adopts a helix–turn–helix conformation, with tandem repeats coalescing into a right‐handed superhelical scaffold (Yin et al., 2013). Functional units typically exist as dimers, with crystallographic studies demonstrating modular recognition of single‐stranded RNA through sequence‐specific interactions (Ban et al., 2013; Yin et al., 2013; Gully et al., 2015; Shen et al., 2016). In PPR proteins, target RNA recognition is mediated by a modular “PPR code” in which each motif binds a specific nucleotide via key amino acid residues at the fifth and last positions (Barkan et al., 2012; Yagi et al., 2013). The correlation between PPR codes and RNA bases was systematically explored and delineated, and a web server PPR code was developed, providing a platform for target RNA prediction and artificial PPR manipulation (Barkan et al., 2012; Yagi et al., 2013; Yan et al., 2019). The interaction between certain PPR proteins and RNA is tight and sufficiently persistent to protect the bound RNA from nucleases, leading to the accumulation of RNA footprints (Ruwe and Schmitz‐Linneweber, 2012). Determining RNA footprint abundance has provided clues regarding the specific activity of individual PPR proteins (Ruwe and Schmitz‐Linneweber, 2012; Ruwe et al., 2016). However, it should be noted that the tandem repeats of PPR motifs themselves, rather than specific amino acid positions, can also play a dominant role in RNA processing by mediating protein interactions (Huang et al., 2015; Song et al., 2024).

## MULTIFUNCTIONAL ROLES OF PPR PROTEINS IN CROP BIOLOGY

In crops, PPR proteins serve as pivotal multifunctional regulators, and their core functions are predominantly manifested in four key dimensions (Figure 1B). First, in seedling growth regulation, these proteins are indispensable for sustaining normal seedling development, as evidenced by the chlorotic phenotypes observed in many *ppr* mutants. Second, in seed development, PPR proteins are critical for seed development, as many *ppr* mutants give rise to defective seeds characterized by arrested embryo and endosperm development. Third, in the restoration of fertility in cytoplasmic male sterile (CMS) crops, PPR function as Restorer‐of‐fertility (Rf) genes. In CMS crop lines, the introduction of PPR‐encoding Rf genes reverses pollen sterility to fertility and restores reproductive capacity. Fourth, in the modulation of stress responses, PPR proteins mediate crop adaptation to both biotic and abiotic stresses. For instance, some *ppr* mutants exhibit thermo‐sensitive chlorotic phenotypes, while others display enhanced resistance to pathogens and tolerance to salt stress. This article comprehensively reviews recent advances in the functional studies of PPR proteins in these processes in crops and highlights emerging insights into their potential applications.

### PPR proteins regulate crop seedling photosynthesis through chloroplast RNA processing

The foods consumed by humans originate from photosynthesis, a process in which autotrophic plants convert solar energy into chemical energy. All crops rely on this chemical energy as their energy source for growth, development, and reproduction. Chloroplasts serve as the site of photosynthesis, providing a relatively independent space and key components for the photosynthetic process, thus establishing photoautotrophic conditions for plants (Arnon et al., 1954). In addition to photosynthesis, chloroplasts are pivotal in the biosynthesis of vital biomolecules, including amino acids, lipids, and carotenoids (Neuhaus and Emes, 2000; Nelson and Ben–Shem, 2004). The post‐transcriptional processing and translation of photosynthesis‐related genes encoded by the chloroplast genome are crucial for developing and maintaining normal chloroplast function (Small et al., 2023). A plethora of studies indicate that nuclear‐encoded PPR proteins participate in these processes either directly or indirectly, and functional defects in PPR proteins may lead to arrested crop seedling photosynthesis.

**Figure 2:**
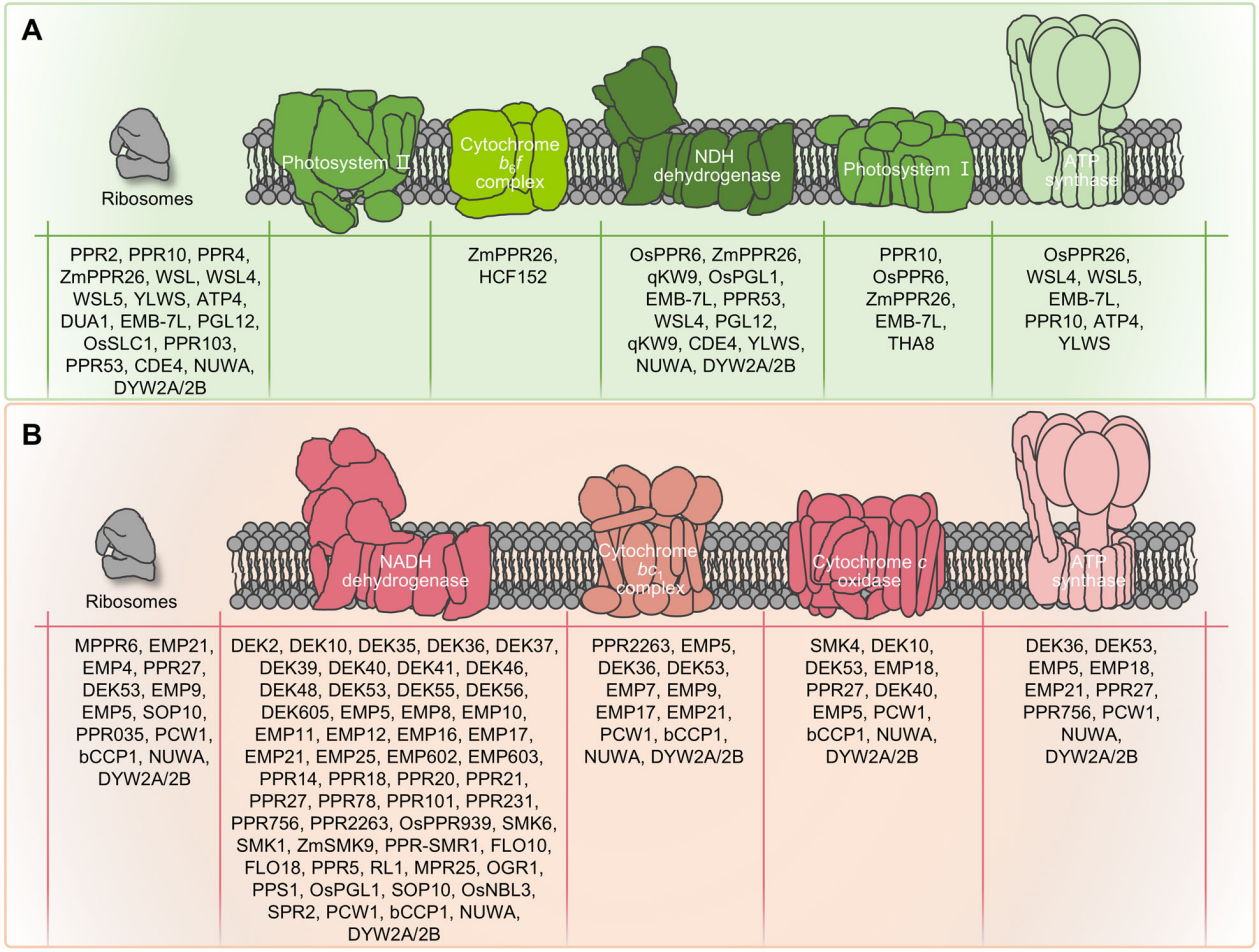
Identified PPR proteins regulating RNA processing of photosynthetic, respiratory, and ribosomal complexes in crop organelles **(A)** PPR proteins associated with RNAs encoding subunits of photosynthetic complexes and ribosomes in chloroplasts. The PPR proteins listed below each complex are implicated in RNA processing for the corresponding complexes. PPR regulators have been identified for ribosomal complexes and all photosynthetic complexes except complex II (for which no PPR regulators have been reported). **(B)** PPR proteins are associated with RNAs encoding subunits of respiratory chain complexes and ribosomes in mitochondria. The PPR proteins listed below each complex are implicated in RNA processing for the corresponding complexes. Among mitochondrial complexes, complex I (NADH dehydrogenase) possesses the most extensively cataloged regulators. This is largely attributed to its structural complexity, which includes a plethora of subunits containing introns and a high density of RNA editing sites.

Maize and rice, two of the world's most important cereal crops, have more than 33 reported PPR proteins localized to their chloroplasts (Table 1). Loss of function in these PPR proteins often leads to a chlorophyll‐deficient phenotype, alterations in chloroplast structure, and seedling lethality. These manifestations are characteristic of photosynthesis‐defective mutants (Figure 1B). The PPR proteins influence chloroplast activities by coordinating the post‐transcriptional processing of organellar RNAs that encode core subunits of chloroplast ATP synthase, chloroplast ribosomes, and photosynthetic complexes (Figure 2A). As currently reported, chloroplast‐localized P‐type PPR proteins primarily process RNAs through RNA stabilization and intron splicing, whereas PLS‐type proteins typically function in chloroplast RNA editing. The corresponding RNA targets for these proteins are presented in Table 1.

**Table 1:** Identified PPR genes across major crops

Crops	Subcellular localization	Gene symbol	Locus ID	Type	Targets	Function	Characterized phenotypes	References
Maize	Chloroplast	*PPR10*	GRMZM2G177169	P	*atpI‐atpH*, *psaJ‐rpl33*	RNA stabilization	Yellow‐green seedling	(Pfalz et al., 2009; Prikryl et al., 2011; Zhelyazkova et al., 2012)
Maize	Chloroplast	*ZmHCF152*	GRMZM2G050697	P	*psbH‐petB*	RNA stabilization	NR	(Zhelyazkova et al., 2012)
Rice	Chloroplast	*OsHCF152*	LOC_Os11g01210/LOC_Os12g01210	P	*psbH‐petB*	RNA stabilization	Pale‐green leaves and seedling lethality	(Mao et al., 2011; Zhelyazkova et al., 2012)
Maize	Chloroplast	*PPR2*	GRMZM2G341621	P	16S, 5S, 4.5S, and 23S rRNA	RNA stabilization	Albino seedling	(Williams and Barkan, 2003)
Maize	Chloroplast	*PPR53*	GRMZM2G438524	PPR‐SMR	23S rRNA	RNA stabilization	Chlorotic seedling	(Zoschke et al., 2016)
Maize	Chloroplast	*PPR5*	GRMZM2G025409	P	*trnG‐UCC* precursor	RNA stabilization	Albino seedling	(Beick et al., 2008)
Maize	Chloroplast	*THA8*	ZEAMMB73_Zm00001d008641	P	*ycf3‐2*, *trnA*	RNA splicing	Pale green leaves and seedling‐lethal	(Khrouchtchova et al., 2012; Ke et al., 2013)
Maize	Chloroplast	*PPR103*	GRMZM2G170896	PPR‐DYW	*rpl16‐rpl14*	RNA stabilization	Albino seedling	(Hammani et al., 2016)
Maize	Chloroplast	*PPR4*	LOC100302579	RRM‐PPR	*rps12*	RNA splicing	Albino seedling	(Schmitz‐Linneweber et al., 2006; Lee et al., 2019)
Rice	Chloroplast	*WSL*	LOC_Os01g37870	P	*rpl2*	RNA splicing	Chlorotic striations, white‐striped leaves	(Tan et al., 2014)
Rice	Chloroplast	*WSL4*	LOC_Os02g35750	P	*atpF, ndhA, rpl2*, and *rps12*	RNA splicing	White‐striped leaves	(Wang et al., 2017b)
Maize	Chloroplast	*EMB‐7L*	GRMZM2G158452	P	*rps12, rpl2, atpF, ndhA, ndhB, rps16* and *ycf3*	RNA splicing	Arrested embryo development and albino seedling	(Yuan et al., 2019)
Rice	Chloroplast	*OsSLC1*	LOC_Os06g49670	P	Multiple group II introns	RNA splicing	Seedling‐lethal chlorosis	(Lv et al., 2020)
Rice	Chloroplast	*ALS3*	LOC_Os01g48380	P	Genes associated with plastid translation and photosynthesis	NR	Albino lethal	(Lin et al., 2015)
Rice	Chloroplast	*YSA*	LOC_Os03g40020	P	Chloroplast‐associated genes	NR	Albino seedling	(Su et al., 2012)
Rice	Chloroplast	*OspTAC2*	LOC_Os03g60910	PPR‐SMR	PEP‐dependent and NEP‐dependent genes	NR	Albino and seedling‐lethal	(Wang et al., 2016)
Rice	Chloroplast	*OsPPR1*	LOC_Os09g24680	P	NR	NR	Albinism and lethality	(Gothandam et al., 2005)
Maize	Chloroplast	*PPR8522*	GRMZM5G884466	P	NR	NR	Embryo deviation and albino seedlings	(Sosso et al., 2012a)
Maize	Chloroplast	*ZmPPR26*	GRMZM2G099604	PPR‐DYW	*atpA, ndhF, rpl20, rpl2, rpoC2, petB, rps8*, and *ndhA*	RNA editing	Albino seedling‐lethal	(Liu et al., 2021b)
Rice	Chloroplast/ Mitochondrion	*OsPGL1*	LOC_Os12g06650	PPR‐DYW	*ndhD* and *ccmFc*	RNA editing	Pale‐green leaves	(Xiao et al., 2018a)
Rice	Chloroplast	*OsPPR6*	LOC_Os05g49920	PPR‐DYW	Editing of *ndhB* and splicing of *ycf3*	RNA editing, RNA splicing	Albino and seedling‐lethal	(Tang et al., 2017)
Rice	Chloroplast	*OsSLA4*	LOC_Os07g07620	PPR‐DYW	*atpF, ndhA, petB, rpl2, rpl16, rps12‐2*, and *trnG*	RNA splicing	Albino and seedling‐lethal	(Wang et al., 2018)
Rice	Chloroplast	*PGL12*	LOC_Os12g10184	PLS	16S rRNA and *ndhA*	RNA splicing	Yellow‐green seedling	(Chen et al., 2019a)
Maize	Chloroplast	*PPR647*	GRMZM2G093098	P	Multiple chloroplast genes	RNA splicing, RNA editing	Albino seedling‐lethal	(Zhao et al., 2021)
Maize	Chloroplast	*ATP4*	GRMZM2G128665	PPR‐SMR	*atpB/E, rps8*	RNA translationRNA editing	Pale‐green chlorotic	(Zoschke et al., 2012; Zhang et al., 2020a)
Maize	Chloroplast	*qKW9*	Zm00001d048451	PPR‐DYW	*ndhB*	RNA editing	Reduced ear and kernel size	(Huang et al., 2020)
Rice	Chloroplast	*TCD10*	LOC_Os10g28600	P	Chloroplast‐associated genes	RNA transcription	Thermo‐sensitive, albino phenotype at 20°C, but normal phenotype at 32°C	(Wu et al., 2016a)
Rice	Chloroplast	*OsV4*	LOC_Os04g39970	P	Genes associated with ribosomal and plastid translation	NR	Albino phenotype at 20°C, but normal phenotype at 32°C	(Gong et al., 2014)
Rice	Chloroplast	*CDE4*	LOC_Os08g09270	P	*rpl2*, *ndhA*, and *ndhB*	RNA splicing	Albino seedling at 20°C but normal at 32°C	(Liu et al., 2021a)
Rice	Chloroplast	*OsATP4/ OsPPR676*	LOC_Os03g11670	PPR‐SMR	*atpB/E*, *rps8*	RNA translation, RNA editing	Temperature sensitive, albino seedling at 20°C, but turns green at natural field conditions	(Zoschke et al., 2012; Liu et al., 2017a; Zhang et al., 2020a)
Rice	Chloroplast	*YLWS*	LOC_Os03g19650	P	Splicing of *atpF*, *ndhA, rpl2*, *rps12*, and editing of *ndhA*, *ndhB*, *rps14*	RNA splicingRNA editing	Temperature sensitive, albino seedling at 20°C, but turn green at 30°C	(Lan et al., 2023)
Rice	Chloroplast	*DUA1*	LOC_Os09g29825	PPR‐DYW	*rps8*	RNA editing	Temperature sensitive, albino seedling at 19°C, but normal at 30°C	(Cui et al., 2019; Du et al., 2021)
Rice	Chloroplast	*WSL5*	LOC_Os04g58780	P	*rps12*, *rpl2*, and *atpA*	RNA splicing, RNA editing	Temperature sensitive, albino seedling at 20°C, but normal at 30°C	(Liu et al., 2018)
Rice	Mitochondrion	*SOP10*	LOC_Os02g07050	PPR‐E	*nad4* intron 1, *nad5* intron 1, *nad2*, *nad6*, and *rps4*	RNA splicing, RNA editing	Enhance the plant's cold resistance at the seedling stage	(Zu et al., 2023)
Rice	Mitochondrion	*PPR035*	LOC_Os01g46230	PPR‐DYW	*rps4‐*926 and *orfX‐*406	RNA editing	Enhanced tolerance to drought and salt at the seedling stage	(Luo et al., 2022)
Rice	Mitochondrion	*PPR406*	LOC_Os10g30760	PPR‐DYW	*orfX‐*355	RNA editing	Enhanced tolerance to drought and salt at the seedling stage	(Luo et al., 2022)
Rice	Mitochondrion	*OsNBL3*	LOC_Os03g06370	P	*nad5* intron 4	RNA splicing	Enhances disease resistance and tolerance to salt	(Qiu et al., 2021)
Maize	Mitochondrion	*DEK2*	GRMZM2G110851	P	*nad1* intron 1	RNA splicing	Small kernel, endosperm and embryo were delayed	(Qi et al., 2017b)
Rice	Mitochondrion	*FLO10*	LOC_Os03g07220	P	*nad1* intron 1	RNA splicing	Floury endosperm and retarded seedling growth	(Wu et al., 2019)
Maize	Mitochondrion	*EMP603*	GRMZM2G069078	P	*nad1* intron 2	RNA splicing	Arrested embryogenesis and endosperm development, empty pericarp	(Fan et al., 2021)
Maize	Mitochondrion	*EMP11*	GRMZM2G353301	P	*nad1* intron 1, 2, 3, and 4	RNA splicing	Impaired embryo and endosperm development, empty pericarp	(Ren et al., 2017)
Maize	Mitochondrion	*DEK37*	GRMZM2G021319	P	*nad2* intron 1	RNA splicing	Delayed endosperm and embryo development	(Dai et al., 2018)
Maize	Mitochondrion	*EMP10*	GRMZM2G078416	P	*nad2* intron 1	RNA splicing	Empty pericarp kernels are unviable	(Cai et al., 2017)
Maize	Mitochondrion	*PPR20*	GRMZM5G818978	P	*nad2* intron 3	RNA splicing	Display slow embryo and endosperm development	(Yang et al., 2020b)
Maize	Mitochondrion	*EMP16*	GRMZM2G060516	P	*nad2* intron *4*	RNA splicing	Severely arrests embryo and endosperm development, resulting in an empty pericarp	(Xiu et al., 2016)
Maize	Mitochondrion	*EMP12*	GRMZM2G023071	P	*nad2* intron 1, 2, and 4	RNA splicing	Severely arrests embryo and endosperm development, causing embryo lethality	(Sun et al., 2019)
Maize	Mitochondrion	*PPR21*	Zm00001eb153040	P	*nad2* intron 1, 2, and 4	RNA splicing	Arrested in embryogenesis and endosperm development, leading to embryo lethality	(Yang et al., 2024)
Maize	Mitochondrion	*DEK35*	GRMZM2G066749	P	*nad4* intron 1	RNA splicing	Lethal‐seed, small and flat seeds at maturity, along with an empty pericarp at the top	(Chen et al., 2017)
Maize	Mitochondrion	*PPR18*	GRMZM2G438456	P	*nad4* intron 1	RNA splicing	Lethal‐seed seriously impairs embryo and endosperm development, resulting in the empty pericarp	(Liu et al., 2020a)
Rice	Mitochondrion	*RL1*	LOC_Os08g41380	P	*nad4* intron 1	RNA splicing	Defective radicle emergence in embryos and compromised grain filling in endosperms	(Wu et al., 2020)
Maize	Mitochondrion	*DEK41*	GRMZM2G127015	P	*nad4* intron 3	RNA splicing	Defective kernel development	(Zhu et al., 2019)
Rice	Mitochondrion	*PPR5*	LOC_Os05g11700	P	*nad4* intron 3	RNA splicing	Floury endosperm and retarded seedling growth	(Zhang et al., 2021b)
Maize	Mitochondrion/Nucleus	*DEK43*	GRMZM2G127015	P	*nad4* intron 1 and 3	RNA splicing	Defective kernels	(Ren et al., 2020a)
Maize	Mitochondrion	*EMP24*/*EMP602*	GRMZM2G464510	P	*nad4* intron 1 and 3	RNA splicing	Empty pericarp	(Ren et al., 2019b; Xiu et al., 2020)
Maize	Mitochondrion	*EMP25*	GRMZM2G312954	P	*nad5* intron 1, 2, and 3	RNA splicing	Impairs embryo and endosperm development	(Xiu et al., 2020)
Maize	Mitochondrion	*PPR101*	GRMZM2G023999	P	*nad5* intron 1 and 2	RNA splicing	Arrests embryo and endosperm development	(Yang et al., 2020a)
Maize	Mitochondrion	*ZmSMK9*	GRMZM2G420723	P	*nad5* intron 1 and 4	RNA splicing	Delayed endosperm and embryo development	(Pan et al., 2019)
Maize	Mitochondrion	*PPR231*	GRMZM2G018757	P	*nad5* intron 1, 2 and 3, *nad2* intron 3	RNA splicing	Arrests embryo and endosperm development	(Yang et al., 2020a)
Maize	Mitochondrion	*PPR14*	GRMZM2G106384	P	*nad7* intron 1 and 2, and *nad2* intron 3	RNA splicing	Severely arrests the embryo and endosperm development, causing an empty pericarp phenotype	(Wang et al., 2020a)
Maize	Mitochondrion	*EMP8*	GRMZM2G388778	P	*nad1* intron 4, *nad4* intron 1, and *nad2* intron 1	RNA splicing	Empty pericarp kernels impair embryogenesis and endosperm development	(Sun et al., 2018)
Maize	Mitochondrion	*PPR‐SMR1*	GRMZM2G345667	PPR‐SMR	Most mitochondrial group II introns	RNA splicing	Both embryo and endosperm development were severely inhibited	(Chen et al., 2019b)
Maize	Mitochondrion	*SPR2*	GRMZM2G122344	P	Splicing of 15 introns	RNA splicing	Embryogenesis and endosperm development were arrested	(Cao et al., 2022)
Maize	Mitochondrion	*DEK46*	GRMZM2G456114	PPR‐DYW	Editing at a specific position in two *nad7* introns	RNA editing	Defective kernel development	(Xu et al., 2020)
Maize	Mitochondrion	*DEK605*	GRMZM2G114241	PPR‐DYW	*nad1‐*608	RNA editing	Delayed seed development	(Fan et al., 2020)
Maize	Mitochondrion	*EMP17*	GRMZM2G019689	PPR‐DYW	*nad2‐*677 and *ccmFc‐*799	RNA editing	Arrests maize kernel development	(Wang et al., 2021c)
Maize	Mitochondrion/Chloroplast	*DEK39*	GRMZM2G345128	PPR‐E	*nad3‐*247 and *nad3‐*275	RNA editing	Defective kernels, development delay in both endosperm and embryo	(Li et al., 2018b)
Maize	Mitochondrion	*DEK10*	GRMZM2G087226	PPR‐E	*nad3‐*61, *nad3‐*62, and *cox2‐*550	RNA editing	Small and shrunken development of kernels affected seedling growth	(Qi et al., 2017a)
Rice	Mitochondrion	*MPR25*	LOC_Os04g51350	PPR‐E	*nad5*	RNA editing	Growth retardation and pale‐green seedling	(Toda et al., 2012)
Maize	Mitochondrion	*PPR2263*	GRMZM2G103078	PPR‐DYW	*nad5‐*1550 *and cob‐*908	RNA editing	Reduced kernel size and a severely stunted vegetative apparatus	(Sosso et al., 2012b)
Maize	Mitochondrion	*ZmSMK1*	GRMZM2G030148	PPR‐E	*nad7‐*836	RNA editing	Arrest both the embryo and endosperm development	(Li et al., 2014)
Rice	Mitochondrion	*OsSMK1*	LOC_Os11g10740	PPR‐E	*nad7‐*836	RNA editing	Abnormal embryo and endosperm development, resulting in embryo or seedling lethality	(Li et al., 2014)
Maize	Mitochondrion	*DEK36*	GRMZM5G892151	PPR‐E+	*atp4*, *nad7*, and *ccmFn*	RNA editing	Produces small, collapsed kernels and lethal seedlings	(Wang et al., 2017a)
Maize	Mitochondrion	*EMP7*	GRMZM2G041231	PPR‐E	*ccmFn‐*1553	RNA editing	Defective kernels and embryo‐lethal	(Sun et al., 2015; Wang et al., 2023)
Maize	Mitochondrion	*PCW1*	AC218148.2_FG008	PPR–DYW	Required for editing at 102 sites	RNA editing	Arrests embryogenesis and endosperm development	(Wang et al., 2023)
Maize	Mitochondrion/Nucleus	*bCCP1*	GRMZM2G158645	bZIP and coiled‐coil domain‐containing PPR	Required for editing at 66 sites	RNA editing	Arrests embryogenesis and endosperm development	(Wang et al., 2023)
Maize	Mitochondrion	*SMK4*	GRMZM2G459532	PPR‐E	*cox1‐*1489	RNA editing	Small plants, delayed embryo and endosperm development	(Wang et al., 2020b)
Maize	Mitochondrion	*EMP18*	GRMZM2G471348	PPR‐DYW	*atp6*‐635 and *cox2*‐449	RNA editing	Arrests embryo and endosperm development, resulting in embryo lethality	(Li et al., 2019)
Maize	Mitochondrion	*DEK40*	GRMZM2G019538	PPR‐E+	*cox3*‐314, *nad2*‐26, and *nad5*‐1916	RNA editing	Defective kernel development	(Ren et al., 2019a)
Rice	Mitochondrion	*OGR1*	LOC_Os12g17080	PPR‐DYW	*ccmC*, *cox2*, *cox3*, *nad2*, and *nad4*	RNA editing	Opaque and growth retardation	(Kim et al., 2009)
Maize	Mitochondrion	*SMK6*	GRMZM2G010046	PPR‐E+	*nad1‐*740, *nad4L‐*110, *nad7‐*739, and *mttB‐*138	RNA editing	Abnormal embryo and endosperm development	(Ding et al., 2019)
Maize	Mitochondrion	*EMP5*	GRMZM2G060536	PPR‐DYW	*rpl16*, *nad9*, *cox3*, *rps12*, *atp6*, *cob*, *nad1*, and *rpl16*	RNA editing	Abortion of embryo and endosperm development	(Liu et al., 2013)
Maize	Mitochondrion	*EMP21*	GRMZM5G849971	PPR‐DYW	Abolished editing at five sites (*nad7‐*77, *atp1‐*1292, *atp8‐*437, *nad3‐*275 and *rps4‐*870), while reduced at 76 sites in 21 transcripts	RNA editing	Severely arrests embryogenesis and endosperm development	(Wang et al., 2019)
Maize	Mitochondrion	*DEK55*	Zm00001d014471	PPR‐E	*atp1*, *atp8*, *ccmFc*, *ccmFn*, *cob*, *mat‐r*, *nad3*, *nad4*, *nad6*, *nad7*, *rps12*, *rps13*, and *rps3*	RNA editing	Defective kernel, arrested embryo and endosperm development	(Ren et al., 2020b)
Maize	Mitochondrion	*PPR27*	GRMZM2G411786	PPR‐DYW	*ccmFn*‐1357 and *rps3*‐707, and six other sites	RNA editing	Arrests in embryogenesis and endosperm development	(Liu et al., 2020b)
Maize	Mitochondrion	*DEK48*	GRMZM2G017197	PPR‐DYW	*nad3*‐185, *nad3*‐215, and *nad4*‐376, *nad4*‐977 sites and decreases the editing at 11 other sites	RNA editing	Defective kernel, embryo and endosperm development are arrested	(Yang et al., 2022)
Maize	Mitochondrion	*DEK53*	GRMZM2G480380	PPR‐E	Editing at more than 60 sites	RNA editing	Defective kernel development	(Dai et al., 2020)
Maize	Mitochondrion	*DEK56*	Zm00001eb188450	PPR‐E	Altered editing efficiency at 48 editing sites	RNA editing	Defective kernels, arrested development of the embryo and endosperm	(Zang et al., 2024)
Maize	Mitochondrion/Chloroplast	*ZmNUWA*	GRMZM2G074599	PPR‐E+	Decreases the editing at 99 mitochondrial sites and 8 plastid sites	RNA editing	Severely arrests embryogenesis and endosperm development	(Wang et al., 2024)
Maize	Mitochondrion/Chloroplast	*ZmDYW2A*	GRMZM2G017821	PPR‐DYW	Multiple editing sites	RNA editing	Single mutants are normal, but the *zmdyw2a zmdyw2b* mutants are severely arrested in seed development	(Wang et al., 2024)
Maize	Mitochondrion/Chloroplast	*ZmDYW2B*	GRMZM2G073551	PPR‐DYW	Multiple editing sites	RNA editing		(Wang et al., 2024)
Maize	Mitochondrion	*EMP4*	GRMZM2G092198	P	*rps2A*‐*rps2B*, *rps3*‐*rpl16*, and *mttb*	RNA stability	Endosperm is drastically reduced, and the embryo is retarded in its development and unable to germinate	(Gutierrez‐Marcos et al., 2007)
Maize	Mitochondrion	*PPR78*	GRMZM2G070381	P	*nad5* mRNA	mRNA stabilization	Arrests embryo and endosperm development	(Zhang et al., 2017b)
Maize	Mitochondrion	*MPPR6*	GRMZM2G389645	P	*rps3*	RNA maturation and translation initiation	Abnormalities in the transfer cell layer, retardation of embryo development, and a considerable reduction of starch level	(Manavski et al., 2012)
Rice	Mitochondrion	*FLO18*	LOC_Os07g48850	P	5′‐end of *nad5*	RNA maturation	Defects in both reproductive and vegetative development.	(Yu et al., 2021)
Rice	Nucleus	*OsNPPR1*	LOC_Os08g19310	P	Multiple genes	RNA splicing	Floury endosperm and retarded seedling growth	(Hao et al., 2019)
Rice	Nucleus	*OsNPPR3*	LOC_Os03g51840	P	Involved in the regulation of expression levels and splicing of a few genes	NR	Chalked endosperm and seed‐lethal phenotypes	(Xue et al., 2019)
Rice	Cytoplasm	*OsCPPR1*	LOC_Os02g02020	P	*OsGLK1* mRNAs	Degrades *OsGLK1*, binds and cleaves the *OsGLK1* mRNA	Abnormal plastid development in tapetal cells, prolonged tapetal programmed cell death and tapetum degradation, and significantly reduced pollen fertility	(Zheng et al., 2022)
Rice	Mitochondrion	*PPS1*	LOC_Os12g36620	PPR‐DYW	*nad3*	RNA editing	Delayed development and partial pollen sterility, enhanced sensitivity to salinity and ABA stress at the vegetative stage	(Xiao et al., 2018b, 2021)
Rice	Mitochondrion	*PPR756*	LOC_Os12g19260	PPR‐E	*atp6*, *ccmC*, and *nad7*	RNA editing	Abortive pollen development	(Zhang et al., 2020b)
Rice	Mitochondrion	*OsPPR939*	LOC_Os05g19390	P	*nad5* introns 1, 2, and 3	RNA splicing	Partially sterile pollen grains	(Zheng et al., 2021)
Rice	Mitochondrion	*PPR762*	LOC9272629	P	NR	NR	Fertility restoration for RT98‐CMS	(Igarashi et al., 2016)
Rice	Mitochondrion	*Rf1a*	LOC9272629	P	*atp6‐orf79*	RNA stabilization	Fertility restoration for CMS‐BT	(Wang et al., 2006)
Rice	Mitochondrion	*Rf1b*	LOC4349026	P	*atp6‐orf79*	RNA stabilization	Fertility restoration for CMS‐BT	(Wang et al., 2006)
Rice	Mitochondrion	*Rf4*	LOC_Os10g35240	P	*WA352*	RNA stabilization	Fertility restoration for CMS‐WA	(Luo et al., 2013; Tang et al., 2014; Zhao et al., 2023)
Rice	Mitochondrion	*Rf20*	OsR498G0102148900.01	P	Competes with WA352 for binding to COX11	Protein interaction	Fertility restoration for CMS‐WA	(Song et al., 2024)
Rice	Mitochondrion	*Rf5*	LOC_Os10g35436	P	*atp6‐orfH79*	RNA splicing	Fertility restoration for CMS‐HL	(Hu et al., 2012)
Rice	Mitochondrion	*Rf6*	LOC_Os08g01870	P	*atp6‐orfH79*	RNA splicing	Fertility restoration for CMS‐HL	(Huang et al., 2015)
Rice	Mitochondrion	*OsRf19*	ON855493	P	*FA182*	RNA cleavage	Fertility restoration for CMS‐FA	(Jiang et al., 2022)
Maize	Mitochondrion	*PPRK2/Rf3*	Zm00031a017461	P	*orf355‐orf77*	RNA editing, RNA stabilization	Fertility restoration for CMS‐S	(Qin et al., 2021)
Maize	Mitochondrion	*ZmRF5*	Zm00001d007531	P	*atp6c*	RNA cleavage	Fertility restoration for CMS‐C	(Lin et al., 2024)
Wheat	Mitochondrion	*Rf1*	R197.300k_Assembly_Contig_120_1	P	*atp8‐orf279*	RNA cleavage	Fertility restoration for T‐CMS	(Melonek et al., 2021)
Wheat	Mitochondrion	*Rf3*	R0934F.300k_Assembly_Contig_78_1	P	*atp8‐orf279*	RNA cleavage	Fertility restoration for T‐CMS	(Melonek et al., 2021)
Rapeseed	Mitochondrion	*PPR‐B*	LOC108827691	P	*orf138‐atp8*	RNA translation	Fertility restoration for CMS‐Ogu	(Uyttewaal et al., 2008; Wang et al., 2021a)
Rapeseed	Mitochondrion	*Rfp*	KX671969.1	P	*orf224‐atp6*	RNA translation	Fertility restoration for CMS‐Pol	(Liu et al., 2012, 2016b)

*Note*: Identified PPR genes in seedling growth, stress response, seed development, and fertility restoration of CMS across major crops.

Abbreviation: NR, Not Reported.

#### PPR proteins regulate chloroplast RNA stabilization in crops

Chloroplast‐targeted P‐type PPR proteins play key roles in stabilizing RNAs by acting as barriers to exoribonucleases. The well‐studied PPR10 protein binds to intergenic regions in *atpI‐atpH* and *psaJ‐rpl33*, protecting the 5′ and 3′ ends of flanking RNAs from degradation (Pfalz et al., 2009; Zhelyazkova et al., 2012). Similarly, HCF152 in rice and maize binds within *psbH‐petB* to stabilize adjacent RNAs (Zhelyazkova et al., 2012). The maize PPR‐SMR protein PPR53, which is orthologous to Arabidopsis SOT1, stabilizes a precursor of 23S rRNA by binding to a sequence approximately 70 nucleotides upstream of its mature 5′‐end, employing a barrier mechanism analogous to PPR10. While PPR53 and its close paralog ATP4 are predicted to bind similar sequences based on the PPR code, their functions differ significantly. PPR53 stabilizes rRNA precursors, whereas ATP4 interacts with the 5′ UTR of *atpB* mRNA and is required for its translation (Zoschke et al., 2012; Zoschke et al., 2016). Although PLS‐class PPR proteins typically specify RNA editing sites, exceptions exist where they perform stabilization functions. PLS‐type protein PPR103 binds specifically near the 5′ end of processed *rpl16* mRNA. By binding between positions ‐54 and ‐27 upstream of the *rpl16* start codon, PPR103 acts as a molecular blockade to 5′ → 3′ degradation, stabilizing dicistronic *rpl16‐rpl14* transcripts and defining the 5′‐end of processed *rpl16* mRNA (Hammani et al., 2016). This stabilization mechanism mirrors that of P‐type PPR proteins like PPR10, and its target RNA recognition resembles that of PLS‐PPR editing factors.

#### PPR proteins regulate chloroplast intron splicing in crops

In chloroplast intron processing, several PPR proteins have been characterized in regulating specific group II intron splicing. For instance, PPR4 contains 16 PPR motifs and an N‐terminal RRM, binds the first intron of *rps12* pre‐mRNA and is essential for its unique *trans*‐splicing (Schmitz‐Linneweber et al., 2006). Further studies have shown that PPR4 has the same role in Arabidopsis and rice (Lee et al., 2019). Rice OsSLC1 contains 12 PPR motifs and participates in the intron splicing of *rps16* (Lv et al., 2020). WSL contains 14 PPR motifs, belongs to the PLS subfamily, and may act as a splicing factor involved in *rpl2* splicing (Tan et al., 2014). Other examples include PLS PPR proteins OsPPR6, facilitating *ycf3* splicing, and PGL12, involved in splicing 16S rRNA and *ndhA*, respectively, while their binding sites and mechanisms remain unclear (Tang et al., 2017; Chen et al., 2019a).

Certain P‐class PPR proteins affect multiple intron targets, unlike the above‐mentioned proteins that affect one or two introns. Among them, rice P‐class PPR protein WSL4 is essential for chloroplast *ndhA*, *atpF*, *rps12*, and *rpl2* introns splicing (Wang et al., 2017b). CDE4 regulates the cold‐adapted RNA splicing of chloroplast genes *rpl2*, *ndhA*, and *ndhB*, a function dependent on its stabilization mediated by interacting with the guanylate kinase VIRESCENT 2 (Liu et al., 2021a). Another rice PLS PPR protein, OsSLA4, is vital for the splicing of multiple introns, such as *atpF* and *ndhA*, and for maintaining transcript levels of chloroplast ribosomal RNAs and photosynthesis‐related genes (Wang et al., 2018). In maize, EMB‐7L mediates the splicing of multiple plastid transcripts, including *rps12* intron1, *rpl2*, *atpF*, and *ndhA* in chloroplasts. Although PPR code analysis predicts EMB‐7L targets *rps12* intron 1, biochemical validation, both *in vivo* and *in vitro*, is required to definitively identify its target intron(s) (Yuan et al., 2019). For several identified chloroplast‐targeted P‐type proteins, including ALS3, YSA, OspTAC2, OsPPR1, and PPR8522, phenotypic characterizations are available, but their RNA targets and molecular functions remain unknown.

PPR proteins have been reported to participate in chloroplast intron splicing through large ribonucleoprotein particles (RNPs), also referred to as spliceosomes. Maize THA8, an unusually small PPR protein with only four PPR motifs, participates in the splicing of both the *ycf3* intron 2 and *trnA* introns (Khrouchtchova et al., 2012). Structural analyses revealed that the THA8 dimer binds two RNA fragments within *ycf3* intron 2, and that RNA binding induces THA8 dimerization (Ke et al., 2013). It has been shown that THA8 functions in large RNPs alongside splicing factors like WTF1 and RNC1, strongly enhancing *trnA* splicing, and its function is conserved between dicots and monocots (Khrouchtchova et al., 2012). THA8 did not significantly coimmunoprecipitate with the chloroplast splicing factors CAF1, CAF2, or CRS1 (Khrouchtchova et al., 2012). However, PPR5 functions within a complex containing the essential splicing factor CAF1 and potentially RNC1. It binds with high affinity to the central region of the *trnG*‐UCC intron and is required for the synthesis or stabilization of the *trnG*‐UCC precursor (Beick et al., 2008). The significantly weaker coprecipitation of PPR5 with RNC1 suggests that PPR5 and RNC1 do not always associate simultaneously with the *trnG*‐UCC intron RNA.

#### PPR proteins regulate chloroplast RNA editing in crops

PPR‐E + and PPR‐DYW proteins are essential for C‐to‐U editing in chloroplast RNA and regulate seedling growth; examples include ZmPPR26, OsPGL1, and OsPPR6 (Tang et al., 2017; Xiao et al., 2018a; Liu et al., 2021b). ZmPPR26 primarily ensures efficient editing at the *atpA*‐1148 site, with minor effects at seven other sites, correlating with its binding affinity to their upstream sequences (Liu et al., 2021b). OsPGL1 is a novel chloroplast and mitochondrial dual‐targeted PPR protein that directly binds and completes editing of chloroplast *ndhD*‐878 and mitochondrial *ccmFc*‐543. Its interactions with OsMORF2/8/9 indicate function within an editosome complex (Xiao et al., 2018a). OsPPR6, a DYW‐type PPR protein, promotes early chloroplast development by participating in the editing of *ndhB* transcripts and the splicing of *ycf3* introns (Tang et al., 2017). It has been proposed that PPR‐DYW proteins typically bind the upstream RNA sequence of editing sites and catalyze the editing process via their DYW domain (Salone et al., 2007; Oldenkott et al., 2019; Takenaka et al., 2021). However, PPR‐E+ proteins function within dynamic editosome complexes, which in plastids may contain additional factors such as MORFs, ORRM1, and OZ1, DYW2A/2B, and NUWA (Wang and Tan, 2024; Wang et al., 2024). In contrast to the involvement of some PLS proteins in intron splicing mentioned above, studies have identified chloroplast P‐type PPR proteins that additionally perform RNA editing alongside their primary roles in RNA splicing, stabilization, and translation. YLWS, a PPR protein containing 11 PPR motifs, directly binds to specific sites on its target RNAs and regulates the splicing of *atpF*, *ndhA*, *rpl2*, and *rps12* transcripts, as well as the editing of *ndhA*, *ndhB*, and *rps14* transcripts (Lan et al., 2023). WSL5 contains 15 PPR motifs and an RRM at its N‐terminus, affects the splicing of *rpl2* and *rps12‐2*, and also the editing efficiency of *rpl2*‐1 and *atpA*‐1148 (Liu et al., 2018). P‐type PPR protein ATP4 has been demonstrated to have an unanticipated role in editing *rps8*, whose expression is responsible for the cold‐sensitive phenotype of rice *osatp4* mutants (Zhang et al., 2020a). Since ATP4 itself does not contain the domain for C‐to‐U conversion in RNA, it was proposed that ATP4 may disrupt the stem loop of *rps8*, which facilitates the binding of DUA1, a PLS‐type PPR protein that might directly edit *rps8* (Cui et al., 2019; Sun, 2020).

### PPR proteins regulate crop seed development primarily through mitochondrial RNA processing

Crop grain is a reproductive organ and provides humans with staple foods rich in sugar and protein. Mitochondria serve as the bioenergetic nexus of seed cells, providing over 90% of cellular ATP through oxidative phosphorylation. Thus, mitochondrial dysfunction directly affects embryo and endosperm development in the seed (Chan, 2006). Due to the loss of independent processing ability, mitochondrially transcribed RNA editing requires PPR proteins to assist in processing, thus ensuring normal embryo and endosperm development (Small et al., 2023). In maize and rice, over 70 PPR proteins localized in mitochondria have been reported (Table 1). Loss‐of‐function mutations in most of these PPR genes consistently lead to defective seed developmental phenotypes (Figure 1B). These phenotypes include defective kernels (dek), empty pericarp (emp), small kernels (smk), opacity and growth retardation (ogr), and floury endosperms (flo). The underlying cause is impaired mitochondrial respiration due to disrupted RNA processing, triggering an energy crisis. This universally disrupts oxidative phosphorylation, often resulting in upregulation of alternative oxidase (AOX) and other mitochondrial complexes. Consequently, mutants exhibit delayed or severely impaired embryo and endosperm development, manifesting as reduced kernel sizes, embryo lethality, or seedling lethality.

The majority of these PPR proteins fine‐tune mitochondrial bioenergetics by mediating RNA splicing, editing, and stability of transcripts in mitochondria (Figure 1A; Table 1). They act on genes essential for mitochondrial complex activity, including those encoding subunits of NADH dehydrogenase (Complex I), cytochrome *bc*_1_ complex (Complex III), cytochrome *c* oxidase (Complex IV), ATP synthase (Complex V), and ribosomal proteins (Figure 2B). Through this regulation of core mitochondrial complexes, PPR proteins are indispensable for cereal seed development.

#### PPR proteins regulate mitochondrial intron splicing in crops

Mitochondrial PPR proteins act as essential cofactors for splicing degenerate group II introns in crops, thereby playing a crucial role in seed development. Splicing defects predominantly arise from loss‐of‐function mutations in P‐type PPR proteins, which exhibit remarkable specificity for individual introns or subsets. Similar to their roles in chloroplasts, PPR proteins participate in mitochondrial intron splicing through large RNPs, although the identified components of these complexes differ from those in chloroplasts. Based on the functions of identified PPR proteins involved in mitochondrial intron splicing, these splicing‐associated PPR proteins can be classified into three categories: 1. Intron(s)‐specific factors. Most mitochondrial P‐type PPR proteins are dedicated to splicing one or a few introns, often requiring functional collaboration. For example, splicing of *nad1* intron 1 requires maize *DEK2*, *EMP11* and rice *FLO10* (Ren et al., 2017; Qi et al., 2017b; Wu et al., 2019; Wu et al., 2023). *DEK37* and *EMP10* are required for the splicing of *nad2* intron 1 (Cai et al., 2017; Dai et al., 2018). *EMP12*, *EMP16*, and *PPR21* are involved in *nad2* intron 4 splicing. *DEK35* and *PPR18* are involved in *nad4* intron 1 splicing (Chen et al., 2017; Liu et al., 2020a). This functional adaptation compensates for degenerated intronic *cis*‐elements, with PPR proteins acting as *trans*‐acting factors that recognize specific sequences, although the binding sites for some of these proteins remain unidentified. 2. Broad‐core splicing factors. SPR2, a small PPR protein containing only four repeats, is essential for splicing the majority (15 out of 22) of mitochondrial group II introns in maize (Cao et al., 2022). Similarly, PPR‐SMR1, a PPR protein with an SMR domain, is required for splicing most (16 out of 22) of maize mitochondrial introns. Crucially, SPR2 and PPR‐SMR1 physically interact, and this interaction enables them to form the core of a splicing complex necessary for the splicing of 13 specific introns (Cao et al., 2022). Both proteins dynamically interact with other splicing factors and recruit substrate‐recognizing PPR proteins to facilitate splicing of their target intron (Chen et al., 2019b; Wang et al., 2020a; Yang et al., 2024). 3. Multifunctional adapters. Some PPR proteins act as adapters, integrating RNA binding with regulatory complex assembly. EMP603 bridges RNA helicase PMH2, CRM domain protein Zm‐mCSF1, and RAD52‐like protein ODB1 to the splicing complex (Fan et al., 2021). Similarly, PPR14 links the specific factor Zm‐mCSF1 to PPR‐SMR1 and forms a complex essential for *nad2* intron 3 and *nad7* intron 1 and intron 2 splicing (Wang et al., 2020a). Notably, a substantial proportion of PPR proteins simultaneously regulate the splicing of most or all introns within a single gene. For instance, EMP11 regulates the splicing of all introns in *nad1* (Ren et al., 2017). Similarly, DEK43 and EMP24 are required for splicing the introns of *nad4* (Xiu et al., 2020; Ren et al., 2020a), while EMP25, PPR101, ZmSMK9, and PPR231 are involved in splicing most introns of *nad5* (Pan et al., 2019; Xiu et al., 2020; Yang et al., 2020a). PPR14 also regulates the splicing of *nad7* introns (Wang et al., 2020a). These studies suggest that the removal of all introns from the same mitochondrial gene is likely coordinately regulated, and the precursors of all the introns for the same gene may be assembled in a temporally and spatially organized spliceosome complex.

#### PPR proteins regulate mitochondrial RNA editing in crops

RNA editing in plant mitochondria is essential for generating functional transcripts, particularly for genes encoding subunits of the mitochondrial respiratory chain. Similar to the process in chloroplasts, mitochondrial RNA editing is executed by PPR proteins within an editosome complex, which shares key components with its chloroplast counterpart (Sun et al., 2013; Wang et al., 2024). PPR‐DYW proteins, typically containing C‐terminal DYW domains, specifically bind upstream of editing sites, with their DYW domain catalyzing the editing. The mitochondrial PPR‐E+ editosome includes members such as PPR‐E + , DYW2A/2B, NUWA, and MORF1/8 (Wang et al., 2024). In contrast, PPR‐E proteins recognize sequences upstream of target cytosines and recruit atypical DYW‐type proteins like PCW1 to catalyze editing (Wang et al., 2023; Zang et al., 2024). This assembly is facilitated by scaffolding proteins such as bCCP1 (a bZIP‐PPR‐CC protein), which bridges PPR‐E proteins and PCW1 (Wang et al., 2023). Factors such as ZmMORF1/8 enhance interactions within the complex, while proteins like ZmGRP23 facilitate PCW1 recruitment via ZmMORFs. In summary, bCCP1, MORF1, and MORF8 exhibit strong interactions with both PCW1 and the PPR‑E protein, thereby assembling an active editosome responsible for recruiting PCW1 to its target cytidine (Wang et al., 2023). This mechanism represents a primary form of mitochondrial RNA editing in maize, which is functionally analogous to the system in Arabidopsis. Defects in these editing mechanisms impair the mitochondrial respiratory chain, highlighting the critical role of RNA editing in plant energy metabolism and seed development.

#### PPR proteins regulate mitochondrial RNA stability and maturation in crops

Studies in maize and rice demonstrate that specific P‐type PPR proteins localized in mitochondria are crucial for seed development by ensuring the stability and proper maturation of mitochondrial mRNAs. In maize, MPPR6 directly binds to the 5′ UTR of mitochondrial *rps3* mRNA, facilitating its 5′‐end maturation and translation initiation (Manavski et al., 2012). Similarly, rice FLO18 is required for the 5′‐end processing of mitochondrial *nad5* transcripts (Yu et al., 2021). Another maize protein, PPR78, stabilizes mature *nad5* mRNA, and its absence results in dramatically reduced *nad5* levels, impaired Complex I formation, and severely arrested seed development (Zhang et al., 2017b). These studies highlight a conserved mechanism whereby mitochondrial‐targeted PPR proteins ensure precise processing and stability of key mitochondrial transcripts, thereby maintaining mitochondrial function, energy supply, and proper nutrient allocation during grain filling and seed development in cereals. Notably, MPPR6, PPR78, and FLO18 all target 5′ UTRs of critical transcripts. Their binding not only stabilizes the mRNAs but also facilitates accurate 5′‐end maturation, ensuring production of functional transcripts. Importantly, the RNA stabilization mechanism mediated by these PPR proteins is highly conserved across chloroplasts and mitochondria, grounded in the principle of sequence‐specific binding to create a protective barrier.

#### Nuclear‐localized PPR proteins in crop seed development

Nuclear‐localized PPR proteins play critical yet underexplored roles in regulating grain development in crops. In rice, two such proteins, OsNPPR1 and OsNPPR3, have been identified as key regulators of endosperm formation and seed viability (Hao et al., 2019; Xue et al., 2019). OsNPPR1 is localized in the nucleus and influences mitochondrial function by binding to the CUCAC RNA motif, thereby affecting the splicing of nuclear genes involved in mitochondrial processes (Hao et al., 2019). Loss of *OsNPPR1* leads to floury endosperm, reduced starch and amylose content, and delayed seedling growth. Similarly, OsNPPR3 is targeted to the nucleolus and is essential for mitochondrial gene splicing (particularly in *nad1* and *nad2* transcripts) (Xue et al., 2019). Mutations in *OsNPPR3* result in chalky endosperm, defective embryo development, and severe seed lethality. In maize, bCCP1 is dual‐localized to mitochondria and the nucleus. While it facilitates RNA editing at numerous mitochondrial sites, its nuclear function remains to be determined (Wang et al., 2023). These findings reveal that nuclear PPR proteins orchestrate nuclear‐mitochondrial crosstalk through post‐transcriptional regulation of mitochondrial RNA metabolism, thereby ensuring proper seed development.

#### Some chloroplast‐targeted PPR proteins in crop seed development

Functional defects in chloroplast‐localized PPR proteins impact vegetative growth and are also involved in embryo and endosperm development. For instance, null mutants of *EMB‐7L* and *PPR8522* result in embryonic lethality (Sosso et al., 2012a; Yuan et al., 2019), while *qKW9* deficiency disrupts *ndhB* RNA editing, compromising photosynthetic output and grain filling efficiency (Huang et al., 2020). Nevertheless, the molecular mechanisms underpinning these proteins remain incompletely understood. Therefore, cloning and further functional characterization of chloroplast PPR proteins involved in grain filling will help decipher the post‐transcriptional regulatory network that links chloroplast gene expression to seed development.

### PPR proteins regulate CMS fertility restoration in crops

Rapid evolution occurring within the mitochondrial genome frequently produces incompatibility between the nucleus and mitochondria, resulting in CMS and restoration of fertility (Rf) systems (Luo et al., 2013; Chen and Liu, 2014; Raval et al., 2023). CMS‐Rf systems serve as an excellent model to study mitochondrial‐nuclear co‐evolution and interaction in plants, as well as a valuable genetic tool for breeding hybrid vigor in crops, and have contributed significantly to food security worldwide (Luo et al., 2013; Chen and Liu, 2014). CMS is widely deployed in the commercial production of hybrid seeds across major crops. An improved understanding of the genetic basis of CMS fertility restoration is crucial for utilizing crop heterosis, particularly in rice, maize, wheat, and rapeseed.

**Figure 3:**
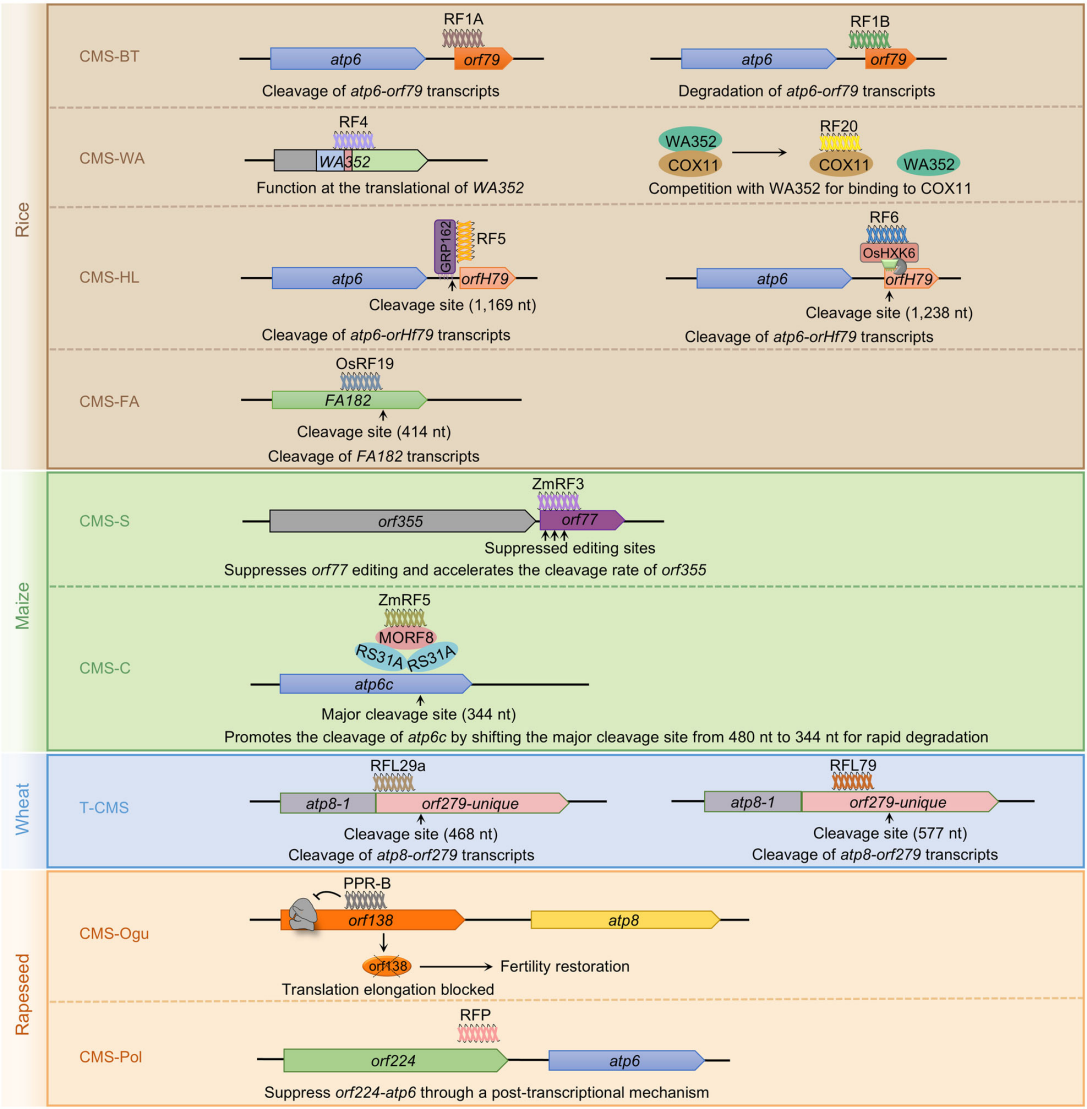
Major cytoplasmic male sterile (CMS) types and their corresponding PPR‐mediated fertility restoration mechanisms in rice, maize, wheat, and rapeseed In rice Boro II CMS (CMS‐BT), the chimeric RNA *atp6‐orf79* is cleaved by PPR protein RF1A and degraded by RF1B, collectively preventing the production of toxic proteins. In rice Wild abortive CMS (CMS‐WA), the PPR protein RF4 directly suppresses the translation of the chimeric RNA *WA352*. Concurrently, RF20 competes with WA352 for binding to COX11, thereby enhancing COX11's function as an ROS scavenger. In rice Honglian CMS (CMS‐HL), the chimeric RNA *atp6‐orfH79* is cleaved by complexes mediated by PPR proteins RF5 and RF6, respectively, to prevent toxic protein translation. In rice Fujian abortive CMS (CMS‐FA), the PPR protein OsRF19 cleaves the chimeric RNA *FA182*, inhibiting toxic protein production. In maize S‐type CMS (CMS‐S), ZmRF3 accelerates cleavage of *orf355* by suppressing RNA editing of *orf77* in the *orf355‐orf77* chimeric transcript. In maize C‐type CMS (CMS‐C), ZmRF5 forms a complex that induces rapid degradation of the *atp6c* transcript through altered cleavage site recognition, thereby effectively blocking the production of the toxic protein. In wheat *Triticum timopheevii*‐type (T‐CMS), the chimeric RNA *atp8‐1*/*orf279*‐unique is cleaved at distinct sites by PPR proteins RFL29a and RFL79, preventing toxic protein expression. In rapeseed Ogura CMS (CMS‐Ogu), PPR‐B blocks the translation elongation of *orf138* in the *orf138‐atp8* chimeric RNA, thus preventing the translation of toxic proteins. In rapeseed Polima CMS (CMS‐Pol), RFP may inhibit the translation of chimeric RNA *orf224‐atp6* via a post‐transcriptional mechanism.

CMS is a maternally inherited trait whereby plants fail to produce viable pollen but maintain female fertility and is widely distributed in higher plants (Chen and Liu, 2014). Typically, it is caused by the expression of novel chimeric open reading frames (ORFs) that result from mitochondrial DNA recombination and are particularly deleterious during pollen development and frequently co‐transcribed with conventional mitochondrial genes. The nuclear‐encoded Rf gene restores pollen production and crop fertility by blocking the expression of chimeric ORFs and reducing the associated harmful effects (Chen and Liu, 2014; Gualberto and Newton, 2017). CMS‐related genes are usually produced during interspecific hybridization, resulting in a new plant carrying heterologous cytoplasm and nucleus (Chase, 2007). In general, the Rf trait is regulated by one or two major genes. The majority of the Rf genes encode PPR proteins. Current research has revealed at least three distinct molecular mechanisms underlying fertility restoration mediated by PPR proteins (Figure 3; Table 1). The first mechanism is the triggering of the cleavage of the chimeric CMS‐associated ORF. This RF protein induces RNA cleavage; however, the exact mechanism remains elusive because Rf proteins lack any known endonuclease motifs. Additionally, the cleavage site is typically located far from the Rf protein binding site. This spatial separation makes it unlikely that the Rf protein directly mediates the cleavage process (Wang et al., 2006; Hu et al., 2012; Liu et al., 2012; Tang et al., 2014; Huang et al., 2015; Melonek et al., 2021; Qin et al., 2021; Jiang et al., 2022; Lin et al., 2024; Song et al., 2024). The second regulatory mechanism involves blocking the ribosome‐mediated translation of the chimeric ORF associated with CMS, which has been characterized in rapeseed (Wang et al., 2021a). The third mechanism involves competing against toxic proteins, thereby enhancing the capacity of reactive oxygen species (ROS) scavenging, which has been identified in rice (Song et al., 2024).

CMS in rice is mediated by three major CMS systems: CMS‐WA, CMS‐BT, and CMS‐HL. Each system exhibits distinct molecular mechanisms underlying CMS and fertility restoration. In the CMS‐BT system, male sterility results from the accumulation of the abnormal mitochondrial protein ORF79 in microspores. This process involves the co‐transcription of mitochondrial *orf79* with the *atp6* gene that encodes subunit 6 of mitochondrial ATPase. Fertility restoration is achieved via the activity of two PPR proteins encoded by the *Rf1* locus: *Rf1a* and *Rf1b*. These mitochondrial‐targeted proteins restore male spore fertility by either cleaving (RF1A) or degrading (RF1B) *atp6‐orf79* messenger RNA, thus preventing ORF79 protein production (Wang et al., 2006). In the CMS‐WA system, the CMS protein WA352 accumulates preferentially in tapetal cells at the microspore mother cell stage and forms a physical interaction with OsCOX11, inhibiting the function of OsCOX11 in peroxidase metabolism, leading to early release of cytochrome *c* and an increase in ROS, resulting in rapid cellular degeneration of the tapetum and subsequent pollen sterility. RF3 acts at the translational or post‐translational level to prevent WA352 protein accumulation without affecting mRNA levels (Luo et al., 2013), while RF4 reduces *WA352* transcript abundance to approximately 20% of normal through an undefined processing mechanism (Luo et al., 2013; Tang et al., 2014). Additionally, RF20 competes with WA352 for COX11 binding, thus interfering with WA352‐COX11 complex‐dependent ROS production and partially restoring fertility (Song et al., 2024). The CMS‐HL system is characterized by an aberrant chimeric mitochondrial transcript, *atp6‐orfH79*, which induces pollen sterility. Fertility restoration is mediated by two nonallelic Rf genes, *Rf5* and *Rf6* (Hu et al., 2012; Huang et al., 2015). These genes function through a complex restoration mechanism involving multiple protein interactions. RF5 forms a restoration of fertility complex (RFC) with GRP162, a Gly‐rich protein that binds to *atp6‐orfH79* through an RNA recognition motif (Hu et al., 2012). The RFC complex, ranging from 400 to 500 kDa, includes RFC3, a WD40‐like repeat protein containing a DUF1620 domain that acts as an essential scaffold for complex assembly (Qin et al., 2016). Unlike RF5, RF6 operates through a distinct pathway, associating with hexokinase 6 (OsHXK6) in mitochondria to promote the processing of the aberrant *atp6‐orfH79* transcript at nucleotide 1238, ensuring normal pollen development (Huang et al., 2015). Notably, RF6 harbors a unique duplication of PPR motifs (absent in rf6 or other known RF proteins) that does not directly interact with OsHXK6 but is indispensable for enabling the RF6‐OsHXK6 interaction and efficient processing of the *atp6‐orfH79* transcript. Recently, a type of Fujian Abortive cytoplasmic male sterility (CMS‐FA) has been identified. CMS‐FA is caused by the chimeric open reading frame *FA182* in the mitochondrial genome, with fertility restoration mediated by the PPR protein OsRF19 (Jiang et al., 2022). OsRF19, RF1A, and RF5 share a common ancestor, and all employ transcript cleavage as a restoration strategy. However, they have diverged to target distinct RNA substrates. RF1A and RF5 cleave homologous *atp6‐orf79* and *atp6‐orfH79* chimeric RNAs at different sites, respectively, while OsRF19 cleaves *FA182* transcripts. The RT98 CMS line RT98A and fertility restoration line RT98C contain cytoplasm from wild rice (*O. rufipogon*) and a nuclear genome from cultivated rice Taichung 65. Through gene mapping and transgenic complementation experiments, it has been shown that the PPR gene *PPR762* is an Rf gene in RT98 CMS (Igarashi et al., 2016). *PPS1* (Xiao et al., 2018b), *PPR756* (Zhang et al., 2020b), and *OsPPR939* (Zheng et al., 2021) function in RNA metabolism of NADH dehydrogenase complex I subunits, their roles appear to be unrelated to endosperm development but specifically associated with the regulatory mechanisms governing pollen development. Recently, it has been demonstrated that the rice cytoplasmic‐localized PPR protein OsCPPR1 contributes to pollen development by directly binding to the single‐stranded regions of *OsGLK1* mRNAs to downregulate its transcription, thereby regulating plastid development and programmed cell death in tapetal cells. This reveals a novel function of cytosolic PPRs and a new regulatory mechanism for rice pollen development (Zheng et al., 2022).

Three major types of CMS have been identified in maize and are designated as CMS‐C, CMS‐T, and CMS‐S based on their distinct mitochondrial DNA genotypes and corresponding nuclear restorer genes (Chase, 2007). The CMS‐S type is associated with the transcript *orf355‐orf77*, which induces male sterility (Xiao et al., 2020). Fertility restoration in CMS‐S is mediated by the mitochondria‐targeted PPR protein PPRK2 (RF3) that binds to *orf77*, suppresses its editing and degradation, and accelerates *orf355* cleavage through an unknown mechanism, ultimately restoring fertility (Qin et al., 2021). In CMS‐C, male sterility is regulated by the chimeric mitochondrial gene *atp6c*, with fertility restoration achieved by the nuclear PPR gene *ZmRf5* (Lin et al., 2024). ZmRF5 localizes to mitochondria and recruits the splicing factor RS31A through MORF8 to form a cleavage complex. This complex shifts the primary cleavage site of *atp6c* transcripts from the 480th nucleotide to the 344th nucleotide, promoting rapid degradation while preserving an optimal amount of *atp6c* RNA for protein translation and ensuring adequate ATP6C production for assembling mitochondrial complex V, thus restoring male fertility.

In wheat, a hexaploid crop with a large and complex genome, the molecular mechanisms of CMS and Rf are less well‐understood compared with rice and maize. The T‐CMS type, derived from Timopheevi wheat cytoplasm, is the most stable and widely used CMS system in wheat. Two key fertility restorer genes, *Rf1* (located on chromosome 1A) and *Rf3* (located on chromosome 1B), have been identified through positional cloning and selective sequence capture (Melonek et al., 2021). Both genes encode PPR proteins containing 20 characteristic PPR motifs, with RF1 (designated RFL79) and RF3 (designated RFL29a) exhibiting 89% nucleotide sequence identity and 83% amino acid similarity. Despite their high sequence similarity and analogous capacity to restore pollen fertility, their target sites are distinct. RFL29a and RFL79 bind to the *orf279* transcript and cleave the RNA at different sites, leading to reduced accumulation of the Orf279 protein in both cases, thus restoring the T‐CMS male fertility. Notably, pyramiding these two Rf genes through breeding strategies shows potential to achieve more robust and environmentally stable fertility restoration.

Two forms of male‐sterile cytoplasm, designated CMS‐Pol and CMS‐Ogu, are observed in most rapeseed varieties. The CMS‐Pol system is associated with the presence of a chimeric open reading frame *orf224‐atp6* (An et al., 2014). RFP, a mitochondria‐targeted PPR protein, may reduce *orf224‐atp6* through a post‐transcriptional mechanism to restore male sterility (Liu et al., 2016b). The CMS‐Nap cytoplasm confers male sterility on a limited number of cultivars that lack the corresponding restorer gene, *Rfn*. Map‐based cloning reveals that *Rfn* encodes a mitochondria‐targeted PPR protein, and it may play a more complicated role in restoring the CMS‐Nap (Liu et al., 2017b). The sterile gene *orf138* of CMS‐Ogu encodes a mitochondrial membrane protein, and the accumulation of this protein leads to male infertility (Bonhomme et al., 1992). The mitochondrial localization protein PPR‐B encoded by the restorer gene *Rfo* can suppress the accumulation of ORF138 protein in the anther tapetum at the post‐transcriptional level and restore pollen fertility (Uyttewaal et al., 2008; Wang et al., 2021a). The RFO/PPR‐B protein suppresses the translation of the CMS transcript by blocking ribosomes, thus repressing the expression of *orf138*.

In addition to the *B. napus* described above, certain dicotyledonous crops possess PPR genes responsible for restoring fertility in CMS. Specifically, Glyma.09G171200 has been validated as a candidate gene for another *Rf3* gene associated with CMS‐RN (Sun et al., 2022). Moreover, *GmPPR576* (Glyma.16G161900) is a candidate Rf gene for the CMS‐N8855 type (Wang et al., 2021b). In cotton, there are two primary CMS systems, CMS‐D2‐2 and CMS‐D8. The genes *Rf1* and *Rf2* play crucial roles in restoring fertility for CMS‐D2 and CMS‐D8, respectively (Feng et al., 2021; Zhang et al., 2023b). PPR genes were clustered on the *Rf1* and *Rf2* loci, and these PPR clusters were associated with their fertility restoration (Yang et al., 2010; Gao et al., 2022). The cloning of Rf genes can facilitate their molecular exploration in crop heterosis and improve the efficiency of the three‐line hybrid breeding system.

### PPR proteins regulate stress response in crops

A multiplicity of PPR proteins have been implicated in crop responses to diverse biotic and abiotic stresses. Chloroplast‐associated PPR proteins exhibit distinct functions and crucial roles in cold stress responses. Mutants such as *osatp4* (Zhang et al., 2020a), *cde4* (Liu et al., 2021a), *dua1* (Cui et al., 2019; Du et al., 2021), *ylws* (Lan et al., 2023), *tcd10* (Wu et al., 2016a), and *osv4* (Gong et al., 2014) display temperature‐dependent phenotypes, showing leaf yellowing and chlorosis, chloroplast abnormalities, or even lethality at low temperatures, but exhibit a return to a normal phenotype at elevated temperatures. The P‐class protein CDE4 participates in the splicing of the *rpl2*, *ndhA*, and *ndhB* introns. YLWS, in addition to participating in the splicing of *atpF*, *ndhA*, *rpl2*, and *rps12*, is also involved in the editing of *ndhA*, *ndhB*, and *rps14*. Other proteins with similar dual functions include WSL5 and OsATP4, with OsATP4 participating in both RNA translation and RNA editing. Mutations in TCD10 and OsV4 alter expression of chloroplast‐associated genes, particularly those associated with ribosomal functions and plastid translation.

Notably, deficiencies in plastid‐localized pseudouridine synthase OsPUS1 affect the biosynthesis of chloroplast ribosomes, resulting in ROS accumulation and low‐temperature albino seedlings. In comparison, a mutation in *SOP10* that encodes a PPR protein for mitochondrial localization can lead to defects in mitochondrial complex I, thus decreasing ROS production and rescuing the *ospus1* albino phenotype. Moreover, the mutation of *SOP10* in *indica* rice varieties can enhance plant cold resistance by reducing ROS levels (Wu et al., 2023). PPR035 and PPR406 influence rice drought and salt tolerance by regulating mitochondrial RNA editing, but they have no noticeable effect on the vegetative growth of rice (Luo et al., 2022). The *pss1‐RNAi* lines exhibit increased sensitivity to salt and ABA stress during the vegetative period, and the PPS1 protein plays a role in the ROS signaling network, assisting in improving tolerance to abiotic stresses (Xiao et al., 2021). *OsNBL3* encodes a mitochondrially localized PPR protein, whose mutation leads to a spontaneous cell death response and H_2_O_2_ accumulation, and its disruption causes a lesion mimic phenotype with several upregulated defense‐related genes, enhancing rice disease resistance and salt tolerance (Qiu et al., 2021). While these studies link PPR genes to stress adaptation and disease resistance in crops, the precise mechanisms behind these associations have not been fully elucidated and represent an important avenue for future research.

## PPR PROTEINS AS MULTIFUNCTIONAL REGULATORS AND EMERGING BIOTECHNOLOGICAL TOOLS IN CROPS

### Unresolved issues of the PPR protein family and future research directions

The PPR family genes, pivotal regulators of RNA metabolism in plant organelles, continue to attract significant research attention because of their multifaceted roles in crop improvement. Current evidence demonstrates that PPR proteins participate in crop photosynthetic efficiency and energy metabolism through their functions in mitochondria and chloroplasts. Meanwhile, mitochondrial‐localized PPR proteins mediate CMS restoration in pollen. Elucidating the molecular interplay between PPR‐mediated organellar RNA processing and phenotypic traits will establish a foundation for biotechnological enhancement of photosynthetic efficiency, grain filling, heterosis utilization, and stress resilience. With the advancement of modern sequencing technology, the genomes of an increasing number of species are being sequenced and analyzed, and the identification of PPR families and genes is experiencing explosive growth, as reported in cotton, millet, wheat, soybeans, poplar, and rapeseed (Zhang et al., 2017a; Li et al., 2018a; Su et al., 2019; Han et al., 2020; Subburaj et al., 2020; Zhang et al., 2023a; Xu et al., 2024). In addition, PPR proteins participate in regulating fiber development in cotton, fruit development and ripening processes in watermelon, wood formation in poplar, and pathogen resistance in wheat (Subburaj et al., 2020; Wang et al., 2020c; Zhang et al., 2021a; Fu et al., 2024; Huo et al., 2024). The functional mechanism underlying these PPR proteins will be further elucidated and utilized in crops.

Despite significant progress over the past two decades, several key questions regarding PPR proteins remain to be addressed in crops. PPR proteins execute their roles in RNA processing through multi‐protein editosome and spliceosome complexes. However, the precise composition and architecture of these complexes remain poorly defined. A deeper understanding of their assembly and structure is essential to achieve precise manipulation of organellar RNA. Furthermore, the SMR domain of PPR proteins holds promise as a potential RNA manipulation tool and warrants further exploration, characterization, and application in crops. Another issue should focus on elucidating how external environmental factors, particularly temperature, influence the efficiency of RNA splicing or editing mediated by PPR proteins. This direction is crucial given the observed temperature‐dependent phenotypes of multiple PPR mutants, where chloroplast function and plant survival are severely compromised under specific temperature stresses (Liu et al., 2018; Cui et al., 2019; Du et al., 2021; Liu et al., 2021a; Lan et al., 2023). A deeper investigation into the mechanisms by which environmental signals modulate PPR protein activity will significantly advance our understanding of crop adaptation and resilience.

The mechanism by which single PPR proteins affect multiple introns remains unclear. They might recognize a common RNA structure specific to these introns, bind to a shared sequence motif among their target introns, or alternatively, a PPR protein may directly target only one specific intron, with effects on other introns being indirect via associated proteins. It remains to be determined which hypothesis is correct. It has been proposed that diverse chloroplast group II intron RNP complexes exist, containing several splicing factors and a set of introns to facilitate splicing of distinct group II introns. These splicing factors are thought to assist intron folding into a catalytically active structure conducive to splicing by stabilizing the native structure, preventing non‐native structures, or destabilizing misfolded structures (Watkins et al., 2007). Nonetheless, the precise role of these splicing factors in intron folding or sequence recognition during splicing is still ambiguous, largely due to the lack of identified precise and direct binding sites for most splicing factors. While the splicing function of many such proteins has been validated via reverse transcription PCR (RT‐PCR) and reverse transcription quantitative PCR (RT‐qPCR), more biochemical evidence is required to pinpoint their specific intron targets. Identifying the specific binding sites of key splicing factors would greatly enhance our current understanding of their roles in RNA splicing.

### PPR proteins as a platform for programmable RNA targeting and future biotechnological applications

PPR proteins have emerged as a promising platform for precise targeting and manipulation of specific RNA sequences, capitalizing on their modular RNA recognition mechanism and programmable nature. Their core advantage lies in the “one‐repeat‐to‐one‐nucleotide” recognition principle and the “PPR code” determined by key amino acids at positions 5 and the last within each repeat (Barkan et al., 2012; Yan et al., 2019). Critically, a foundational study has demonstrated that plant PPR‐DYW editing factors can perform targeted C‐to‐U editing of nuclear transcripts in human cells (Lesch et al., 2022). This enables rational design and construction of synthetic designer PPR (dPPR) proteins capable of binding to virtually any RNA sequence of interest (Miranda et al., 2018). Unlike CRISPR systems, which require guide RNAs, PPR proteins confer RNA recognition entirely through their polypeptide chains. This intrinsic feature circumvents the challenge of delivering guide RNAs across organellar membranes, presenting a distinct advantage for organelle RNA manipulation.

**Figure 4:**
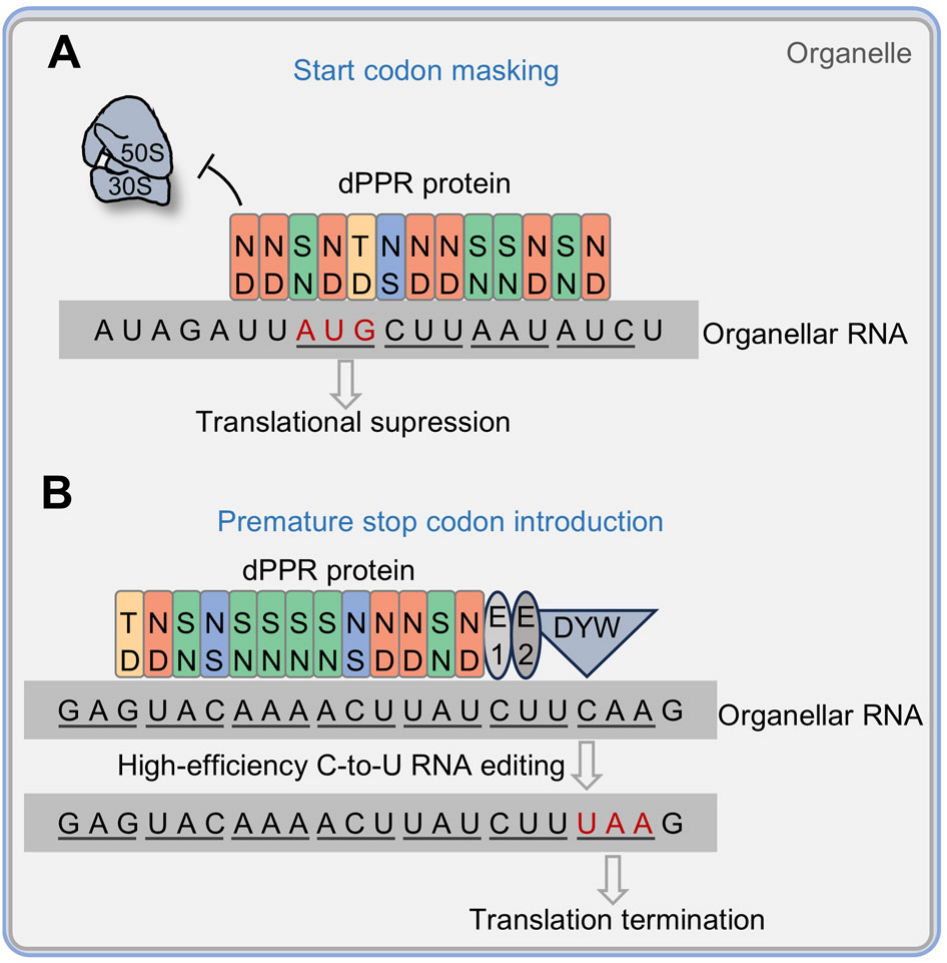
Two mechanisms mediate specific translational repression of organelle mRNAs through designer dPPR proteins **(A)** Start codon masking. By binding near the start codon (e.g., within the sequence AUAGAUUAUGCUUAAUAUCU), the dPPR protein prevents recruitment of the translation initiation complex (Manavski et al., 2025b). **(B)** Premature stop codon introduction. Binding of the dPPR protein to its target sequence (e.g., GAGUACAAAACUUAUCUUCAAG) induces highly efficient RNA editing (up to 85%), introducing a premature stop codon. This leads to translation termination and the production of a truncated polypeptide (Manavski et al., 2025a).

Recent advances in this field have focused on expanding functional applications and overcoming historical technical hurdles. The engineering of novel PPR‐effector fusion proteins has significantly broadened their functional scope (Mathieu et al., 2025; Manavski et al., 2025a, 2025b). Designing dPPR proteins to bind near the start codon of target mRNAs (e.g., *psbK* in chloroplasts and *nad7* in mitochondria) has enabled specific translational suppression, resulting in clear molecular and physiological phenotypes (Figure 4A). It has been confirmed that dPPRs can be used to inhibit translation initiation with high specificity and minimal off‐target effects, as supported by proteomic analyses (Manavski et al., 2025b). Moreover, fusing a programmable PPR RNA‐binding domain with a DYW cytidine deaminase domain has yielded highly efficient, guide‐free C‐to‐U editing tools such as RECODE, which demonstrates high editing efficiency and favorable tissue specificity in human cells and mice (Ichinose et al., 2025). Notably, the designer protein dPPR‐nad7‐DYW, engineered to target a *de novo* site in plant mitochondrial nad7 mRNA, achieved up to 85% editing efficiency, successfully introducing a premature stop codon and creating a stable loss‐of‐function phenotype (Figure 4B). This strongly validates the platform's capability for efficient and precise gene knockdown. Furthermore, the development of modular toolkits like GRASP provides crucial support for these applications by solving the historical challenge of synthesizing and assembling highly repetitive dPPR genes (Dennis et al., 2025). Utilizing Golden Gate assembly, GRASP offers a standardized plasmid library for efficiently constructing dPPR proteins targeting RNAs of varying lengths and supports high‐throughput functional screening, greatly accelerating the design‐build‐test cycle for dPPR proteins. The RNA‐editing approach of dPPR offers a novel and rapid alternative to existing methods, such as mitoTALENs and the more refined systems mitoTALENCD or TALE–DddA. Although mitoTALENs can generate DNA‐level knockouts (Zhou et al., 2024), they pose risks of unintended large deletions and ectopic recombination, and their application necessitates laborious procedures to achieve homoplasmy. The mitoTALENCD system addresses the deletion issue by replacing the nuclease with a cytidine deaminase, enabling precise C‐to‐T conversions (Nakazato et al., 2022). However, attaining homoplasmy with this system still requires a lengthy selection process. Similarly, the TALE–DddA system utilizes an interbacterial toxin to catalyze cytidine deamination within dsDNA (Mok et al., 2020). Nevertheless, it has been suggested that the off‐target effects of this base‐editing tool require thorough definition and evaluation (Lei et al., 2022). In contrast, the PPR‐based system operates at the RNA level, offering a reversible and dynamic means of regulation without altering the mitochondrial genome, and enabling the generation of functional mutants in a significantly shorter timeframe. Beyond these applications in manipulation, engineered PPR proteins also serve as powerful tools for visualizing RNA in living cells. Cellular RNA is not uniformly distributed but organized according to functional needs, a spatial arrangement critical for precise gene regulation. Artificial PPR proteins can be fused to fluorescent tags, such as GFP. These designer proteins bind their target RNA sequences with high specificity, allowing the attached fluorescence to directly report the localization and dynamics of the RNA *in vivo*.

Despite the promising prospects, current PPR protein technology still faces challenges, primarily concerning the further optimization of specificity, efficiency, and delivery. Recent single‐molecule studies have revealed that dPPRs undergo significant conformational compression upon RNA binding and do not rely on scanning non‐target sequences, instead directly recognizing the target sequence, a mechanism that ensures high binding specificity (Marzano et al., 2024). Notably, PPR protein binding is susceptible to RNA secondary structure and sequestration of the target sequence within stable secondary structures can significantly delay or even prevent binding, highlighting the need to avoid stable secondary structures in the target region during design (Marzano et al., 2024). Longer PPR arrays may increase tolerance for mismatches, and nucleotides upstream of the target site (particularly a purine at the −1 position) can inhibit the editing activity of the DYW domain (Miranda et al., 2018; Maeda et al., 2022). Future directions may include employing machine learning to optimize the PPR code and motif design for enhanced specificity, exploring effector domains beyond DYW to diversify functionality, and developing inducible or tissue‐specific expression systems to achieve spatiotemporal control over RNA manipulation.

In summary, PPR proteins have evolved from proof‐of‐concept to practical application as a programmable RNA‐targeting platform. They offer unique advantages in addressing the challenges of organellar gene manipulation, enabling RNA editing, and facilitating the construction of complex synthetic biological circuits. With the continued maturation of design tools, functional modules, and delivery strategies, PPR‐based technologies are poised to open new frontiers in fundamental research, therapeutic development for human diseases, and agricultural biotechnology.

## CONCLUSION

PPR proteins play irreplaceable roles in coordinating nucleus‐organelle communication and regulating key biological processes in crops. This review systematically summarizes the research advances of PPR proteins in major cereal and oilseed crops over the past decade, highlighting their core functions in four critical aspects of crop biology. Chloroplast‐localized PPR proteins mediate RNA stabilization, splicing, and editing to ensure proper chloroplast biogenesis and seedling photosynthesis, while mitochondrial‐targeted PPR proteins regulate RNA splicing, editing, stabilization, and maturation to support embryo and endosperm development. As RF genes, PPR proteins reverse CMS through diverse mechanisms, such as RNA cleavage, translational inhibition, and interference with protein interactions, laying the foundation for hybrid breeding. Additionally, PPR proteins participate in biotic and abiotic stress responses, with temperature‐dependent phenotypes and ROS‐related regulatory effects being prominent features. Notably, the functional mechanisms of PPR proteins exhibit both conservation and specificity across organelles and biological processes. While their core role in regulating organellar RNA processing is conserved across major crops, variations exist in substrate RNAs, regulatory complexes, and modes of action. The emerging dPPR proteins, leveraging the “one‐repeat‐to‐one‐nucleotide” recognition principle and programmable “PPR code”, offer unique advantages in organellar gene manipulation. Their applications in targeted RNA editing, translational suppression, and RNA visualization open new avenues for RNA research and precise crop breeding.

## CONFLICTS OF INTEREST

The authors declare no conflicts of interest.

## AUTHOR CONTRIBUTIONS

M.W., S.Y., and X.Z. designed the study and edited the manuscript. M.W. and M.C. wrote and revised the manuscript. M.C., R.Z., J.Y., G.Z., and F.Y. collected and organized the literature. All authors reviewed and approved the final manuscript.
